# A Novel System for Evaluating the Inhibition Effect of Drugs on Cytochrome P450 Enzymes *in vitro* Based on Human-Induced Hepatocytes (hiHeps)

**DOI:** 10.3389/fphar.2021.748658

**Published:** 2021-10-28

**Authors:** Yan Li, Ying-Yuan Lu, Jun Jia, Meng Fang, Lin Zhao, Yong Jiang, Yan Shi, Peng-Fei Tu, Xiao-Yu Guo

**Affiliations:** ^1^ School of Pharmaceutical Sciences, State Key Laboratory of Natural and Biomimetic Drugs, Peking University, Beijing, China; ^2^ Stem Cell Research Center, School of Basic Medical Sciences, Peking University Health Science Center, Beijing, China

**Keywords:** human induced hepatocytes, cytochrome P450, cocktail, notoginseng, safflower, compatibility

## Abstract

Cytochrome P450 (CYP) is the most important phase I drug-metabolizing enzyme, and the effect of drugs on CYP enzymes can lead to decreased pharmacological efficacy or enhanced toxicity of drugs, but there are many deficiencies in the evaluation models of CYP enzymes *in vitro*. Human-induced hepatocytes (hiHeps) derived from human fibroblasts by transdifferentiation have mature hepatocyte characteristics. The aim was to establish a novel evaluation system for the effect of drugs on CYP3A4, 1A2, 2B6, 2C9, and 2C19 *in vitro* based on hiHeps. Curcumin can inhibit many CYP enzymes *in vitro*, and so the inhibition of curcumin on CYP enzymes was compared by human liver microsomes, human hepatocytes, and hiHeps using UPLC-MS and the cocktail method. The results showed that the IC_50_ values of CYP enzymes in the hiHeps group were similar to those in the hepatocytes group, which proved the effectiveness and stability of the novel evaluation system *in vitro*. Subsequently, the evaluation system was applied to study the inhibitory activity of notoginseng total saponins (NS), safflower total flavonoids (SF), and the herb pair of NS–SF on five CYP enzymes. The mechanism of improving efficacy after NS and SF combined based on CYP enzymes was elucidated *in vitro*. The established evaluation system will become a powerful tool for the research of the effect of drugs on the activity of CYP enzymes *in vitro*, which has broad application prospects in drug research.

## Introduction

Drug-metabolizing enzymes play a key role in the process of drug metabolism (Underhill and Khetani, 2018), especially drug-drug interaction (DDI) in which drug-metabolizing enzymes have a significant impact on the safety of clinical drugs (Akamine et al., 2019). At present, the effect of drugs on metabolic enzymes, especially on the activity of cytochrome P450 (CYP) in phase I metabolism, has been listed as an important part of preclinical research of new drugs by the Food and Drug Administration in many countries (Yu et al., 2019; Sudsakorn et al., 2020). With the increasing development of new drugs, a rapid, accurate, stable, and low-cost evaluation system for the effect of drugs on CYP enzymes has become particularly important.

Liver microsomes are the most widely used in drug metabolism research because of their low cost, simple operation, and rapid detection (Williams et al., 2004; Cheng et al., 2018; Lu et al., 2018). However, liver microsomes are difficult to use when simulating the complete metabolic environment *in vivo* and evaluating the induction ability of drugs on CYP enzymes, so it is hard to accurately and comprehensively reflect the effect of drugs on the activity of CYP enzymes (Diao and Huestis, 2019; Thiengsusuk et al., 2020). Human hepatocytes are the closest model to human liver tissue, which can be used to evaluate the induction and inhibition effect of drugs on CYP enzymes relatively accurately, but human hepatocytes are difficult to obtain, unable to subculture, expensive, and have a short duration for the activity of metabolic enzymes, which is not conducive to extensive application for evaluation of the effect of drugs on CYP enzymes *in vitro* (Zeilinger et al., 2016; Schink and Dehus, 2017; Inoue et al., 2020). In recent years, some evaluation systems of the effect of drugs on CYP enzymes *in vitro* were established based on HepG2, HepRG, and other cells (Darnell et al., 2011; Cui et al., 2014), however, the expression of CYP enzyme activity was not stable in these cells, resulting in poor repeatability and accuracy for evaluation of the effect of drugs on CYP enzymes *in vitro*.

Human-induced hepatocytes (hiHeps), a kind of human hepatocyte model with stable metabolism function, were established through inducing human skin fibroblasts using HNF1A, HNF4A, HNF6, FOXA3, and other cytokines (Du et al., 2014; Huang et al., 2014). A series of phase I and phase II drug-metabolizing enzymes and drug transporters could be expressed in hiHeps, and the metabolic activities of CYP3A4, CYP1A2, CYP2B6, CYP2C9, and CYP2C19 were similar to those of freshly isolated human hepatocytes (Du et al., 2014). The metabolic enzyme activity could be maintained for about 2 weeks, meanwhile, the rapid cell proliferation rate was beneficial to expand the culture. Therefore, hiHeps have an obvious advantage as an evaluation model of related research dependent on CYP enzymes *in vitro* (Du et al., 2014). However, at present, there is no evaluation system of the effect of drugs on CYP enzymes *in vitro* based on hiHeps.

The research used hiHeps as a model to establish a new evaluation system for the effect of drugs on CYP enzymes *in vitro*, which was carried out by determining the Michaelis constant of specific probe substrates of CYP1A2, CYP2B6, CYP2C9, CYP2C19, and CYP3A4, respectively, and investigating the interaction between the probe substrates in the cocktail method. After the establishment of the system, it was compared with the systems of human liver microsomes (HLM) and human hepatocytes to check its validity and stability. Notoginseng and safflower display a synergistic effect in the treatment of cardiovascular disease in clinic and are necessary to explore the mechanism of the synergistic effect of NS and SF through the aspect of metabolism (Meng et al., 2018). Therefore, the system was applied to evaluate the inhibition influences of notoginseng total saponins (NS), safflower total flavonoids (SF), and the herb pair of notoginseng–safflower (namely CNS) on the activity of CYP enzymes. This study aims to set up a novel system based on hiHeps to evaluate the effect of drugs on CYP enzymes *in vitro*, and provide new methods and ideas for drug safety/toxicity research in preclinical and clinical stages.

## Materials and Methods

### Chemicals and Reagents

Nicotinamide adenine dinucleotide phosphate (NADPH), MgCl_2_, phenacetin (PHE), diclofenac (DIC), acetaminophen (ACT), ticlopidine (TIC), thiotepa (THI), sulfaphenazole (SUL), α-naphthoflavone (NAP), alamethicin, and DMSO were purchased from the Sigma–Aldrich Company (St. Louis, United States). Bupropion (BUP), carbamazepine (CAR, internal standard), ketoconazole (KET), and midazolam (MID) were obtained from National Institutes for Food and Drug Control (Beijing, China). Mephenytoin (MEP), hydroxybupropion, 4′-hydroxydiclofenac, 4′-hydroxymephenytoin, and 1′-hydroxymidazolam were gained from the Toronto Research Chemicals Company (Toronto, Canada). Curcumin was obtained from Chengdu Manster Biotechnology Co., Ltd. (Chengdu, China). The purities of all standards were more than 98%. InVitro GRO CP culture medium and fetal bovine serum (FBS) were purchased from Celsis *In Vitro* Technologies Inc (Maryland, United States). HLM (batch number: X008067) and human hepatocytes (batch number: X008000) were purchased from Research Institute for Liver disease Co., Ltd (Shanghai, China). Methanol, acetonitrile, and formic acid of LC-MS grade were obtained from Merck (Darmstadt, Germany). Ultrapure water was produced in the Millipore-Q system (Milford, MA, United States). All the reagents were at least of analytical grade.

NS, consisting of notoginsenoside R1 (6.2%), ginsenoside Rb1 (32.5%), Rd (6.6%), Re (4.1%), and Rg1 (26.6%), was collected by Yunnan Plant Pharmaceutical Co., Ltd (Kunming, China). Safflower, which was provided from Xinjiang Uygur Autonomous Region in China, was authenticated by PFT. The voucher specimen (No. 20110301) was deposited in the Modern Research Center for Traditional Chinese Medicine, Peking University (Beijing, China). The materials of safflower were refluxed with deionized water at 80°C three times (120 L for 1 h, 100 L for 0.5 h, and 100 L for 0.5 h), and the extract was filtered and concentrated in a vacuum. The concentrated solution was subjected to a D101 macroporous resin column eluted with deionized water and 50% aqueous ethanol. The 50% aqueous ethanol eluate was dried by spray drying to obtain SF and the amount of HSYA in SF was more than 8.0% verified by high performance liquid chromatography. CNS was composed of NS and SF with the ratio of 6:5.

### Sample Pretreatment

A total of 250 μL of incubation solution was added to the corresponding 1.5 ml EP tube containing 750 μL of methanol consisting of internal standard (IS). The ice bath was maintained for 60 min after vortex-mixing, and then the solution was centrifuged at 14,000 rpm for 15 min at 4°C. The supernatant was centrifuged twice and 400 μL of the supernatant was evaporated to dryness under reduced pressure at 25°C. The residue was dissolved in 100 μL of 50% methanol (methanol: water, v/v), centrifuged at 14,000 rpm for 15 min, and then the supernatant was injected into the UPLC-MS/MS system for analysis.

### UPLC-MS/MS Conditions

The analysis was conducted on a UPLC ACQUITY H-Class system (Waters, Milford, MA, United States) coupled with an AB SCIEX QTRAP 4500 mass spectrometer (SCIEX, Foster, United States) via an ESI interface. Sample separations were performed on an Acquity UPLC Xbridge BEH C18 column (2.1 mm × 50 mm, 2.5 μm, Waters, Milford, United States) and column temperature was set at 40°C. The mobile phase with the flow rate of 0.6 ml/min consisted of (A) 0.1% formic acid in water and (B) 0.1% formic acid in acetonitrile. Gradient elution condition was as follows: 0–1.5 min, 5% B; 1.5–3.5 min, 5%–45% B; 3.5–8.0 min, 45%–100% B; 8.0–11.0 min, 100% B. The injection volume was 1.0 μL. The ion source parameters of MS were kept as follows: MRM mode; positive ion mode; collisional activated dissociation (CAD) gas, medium level; ion spray needle voltage, 5500 V/−4500 V; GS1, 45 psi; GS2, 45 psi; CUR, 35 psi; and turbo gas temperature, 550°C. Analyst 1.6.2 was used to analyze data in this experiment. The MS parameters of probe drugs and corresponding metabolites are shown in Supplementary Table S1.

The quantitative determination method of probe drug metabolites (acetaminophen, hydroxybupropion, 4′-hydroxydiclofenac, 4′-hydroxymephenytoin, and 1′-hydroxymidazolam) was verified by specificity, linearity, precision, accuracy, recovery, and matrix effects. Calibration curves had to have correlation coefficients (r) of 0.99 or better. The lower limit of quantification (S/N ≥ 10) was expressed as the lowest concentration of the standard curve. Relative standard deviation (RSD) and relative error (RE) were defined as precision and accuracy, which were set to be less than ±15% (for the low concentration, the acceptance criterion was less than 20%). The extraction recoveries of the analytes were calculated by determining the peak area ratios of the analytes in the post-treatment spiked samples to that acquired from pre-treatment spiked samples. The matrix effect was investigated by comparing the peak areas of the analytes dissolved in the pre-treated blank incubation solution with that of the pure standard solutions containing equivalent amounts of the analytes.

### Establishment of an Evaluation System for the Effect of Drugs on the Activity of Cytochrome P450 Enzymes *in vitro* Based on Human-Induced Hepatocytes

#### Determination of Michaelis Constant of Five Cytochrome P450 Enzymes in Human-Induced Hepatocytes

The hiHeps were digested with accutase; the cell-survival rate was detected with trypan blue staining, and counted with hemocytometer measurement. In total, 2×10^5^ cells per milliliter of culture medium was used as the cell suspension. The standard working solutions of probe drugs were prepared by a spiking incubation solution to give the nominal concentrations of 20, 40, 80, 120, 160, and 200 μM for PHE (CYP1A2, substrate), 50, 100, 200, 400, 600, and 1000 μM for BUP (CYP2B6, substrate), 12.5, 25, 50, 100, 200, and 400 μM for DIC (CYP2C9, substrate), and 5, 10, 20, 50, 100, and 200 μM for MEP (CYP2C19, substrate) and MID (CYP3A4, substrate). A 250 μL cell suspension and 250 μL probe incubation solution were mixed in a 5 ml flow tube, which was placed in a 37°C shaker and incubated for 60 min. The samples were pretreated according to “Sample Pretreatment.”

#### Study on the Interaction Between Probe Drugs

The standard working solutions of probe drugs were prepared by diluting the stock solution to obtain the nominal concentrations of 125 μM for PHE and BUP, 50 μM for DIC and MEP, and 25 μM for MID. Serial dilutions of NAP (CYP1A2 inhibitor, 0, 0.001, 0.005, 0.01, 0.05, and 0.1 μM), THI (CYP2B6 inhibitor, 0, 0.1, 1, 10, 50, and 100 μM), SUL (CYP2C9 inhibitor, 0, 0.05, 0.5, 5, 50, and 500 μM), TIC (CYP2C19 inhibitor, 0, 0.05, 0.5, 5, 50, and 500 μM), KET (CYP3A4 inhibitor, 0, 0.005, 0.01, 0.05, 0.1, and 1 μM), and cocktail solution that included the above drugs were prepared for use. A 250 μL cell suspension, 200 μL probe incubation solution, and 50 μL inhibitor incubation solution were mixed in a 5 ml flow tube, which was placed in a 37°C shaker and incubated for 60 min.

### Validation of System for the Effect of Drugs on the Activity of Cytochrome P450 Enzymes *in vitro* Based on Human-Induced Hepatocytes

#### Inhibitory activity of Curcumin on Five Cytochrome P450 Enzymes in Human Liver Microsomes

Human liver microsomes (HLM, 20 mg/ml) were added to an equal volume of Tris-HCl and double volume of 1 mg/ml of macromolecular surfactant (Brij), which had no effect on the activity of CYP enzymes, and were activated by ice bath for 5 min. The activated HLM were added into the corresponding channel of 96-well plates, then MgCl_2_ and alamethicin were added, and an ice bath was used for 20 min for reactivation. The mixed solution of probe drugs consisting of 50 μM PHE, 50 μM BUP, 20 μM DIC, 20 μM MEP, and 10 μM MID and serial dilutions of curcumin (0, 1, 10, 50, 100, and 500 μM) were added to the reaction system. The reaction system was pre-incubated at 37°C for 20 min, then NADPH was added, and incubated at 37°C for 60 min. The samples were pretreated according to “Sample Pretreatment.”

#### Inhibitory activity of Curcumin on Five Cytochrome P450 Enzymes in Human Hepatocytes

In total, 45 ml of InVitroGro CP culture medium (without FBS) was preheated to 37°C, and then 5 ml of FBS and 1 ml of Torpedo Antibiotic Mix was added. Then, 5 ml of InVitroGro CP culture medium was transferred to a 50 ml sterile conical tube. Human hepatocytes were transferred into the preheated InVitroGro CP culture medium and the tube was gently reversed three times to suspend hepatocytes again. The cell-survival rate was detected by trypan blue staining and counted by hemocytometer measurement. Overall, 2 × 10^5^ cells per milliliter of culture medium was used as the cell suspension. The remaining treatments were the same as the method described in the “Inhibitory activity of curcumin on five CYP enzymes in human liver microsomes” section.

#### Inhibitory activity of Curcumin on Five Cytochrome P450 Enzymes in Human-Induced Hepatocytes

The hiHeps to be detected were digested with accutase, the cell-survival rate was detected by trypan blue staining, and counted with hemocytometer measurement. A total of 2 × 10^5^ cells per milliliter of culture medium was used as the cell suspension. The remaining treatments were the same as the method described in the “Inhibitory activity of curcumin on five CYP enzymes in human hepatocytes” section.

### Application of System for Cytochrome P450 Enzyme Activity *in vitro* Based on Human-Induced Hepatocytes

#### Inhibitory activity of CNS, NS, and SF on Five Cytochrome P450 Isoenzymes in Human-Induced Hepatocytes

The treatment of hiHeps was the same as the method described in the section of “Inhibitory activity of curcumin on five CYP enzymes in hiHeps.” The stock solutions of SF, NS, and CNS were diluted to provide incubation solutions (5 mg/ml, 6 mg/ml, and 11 mg/ml), respectively. A 250 μL cell suspension, 200 μL incubation solution of probe drugs, and 50 μL incubation solution of SF, NS, or CNS were mixed in a 5 ml flow tube, placed in a 37°C shaker, and incubated for 60 min. The samples were pretreated according to “Sample Pretreatment.”

### Enzyme Kinetic Parameters and Statistical Analysis

The kinetic parameter Michaelis constant (K_m_) of the five probe drugs was estimated by nonlinear regression from a Lineweaver–Burk plot on the basis of the Michaelis–Menten equation, 1/v = 1/V_max_+(K_m_/V_max_)×1/[S], where v is the rate of reaction and [S] is the substrate concentration. Drug inhibition was calculated on the basis of the metabolite response observed in the presence of a test drug to the vehicle control. Non-linear fitting and determination of IC_50_ values were calculated using IBM SPSS Statistics 20.0. The data were expressed as the mean ± standard deviation (SD). Statistical analysis was determined by Student’s t-test with a two-tailed distribution and one-way analysis of variance (ANOVA), and a *p* value below 0.05 was considered statistically significant.

## Results and Discussion

### Method Validation

There was no interference observed from endogenous substances in the samples at the retention time of acetaminophen, hydroxybupropion, 4′-hydroxydiclofenac, 4′-hydroxymephenytoin, 1′-hydroxymidazolam, and IS (Supplementary Figure S1). We detected the standard curve, linear range, correlation coefficient, and lower limit of quantitation (LLOQ) for each metabolite of probe substrate by UPLC-MS (Supplementary Table S2). The intra-day and inter-day precisions were less than 5.21% and accuracies changed from 98.55 to 102.54% (Supplementary Table S3). The matrix effects and extraction recoveries were more than 78.59 and 86.36%, respectively (Supplementary Table S4).

### Determination of Michaelis Constant for the Five Probe Drugs

**FIGURE 1:**
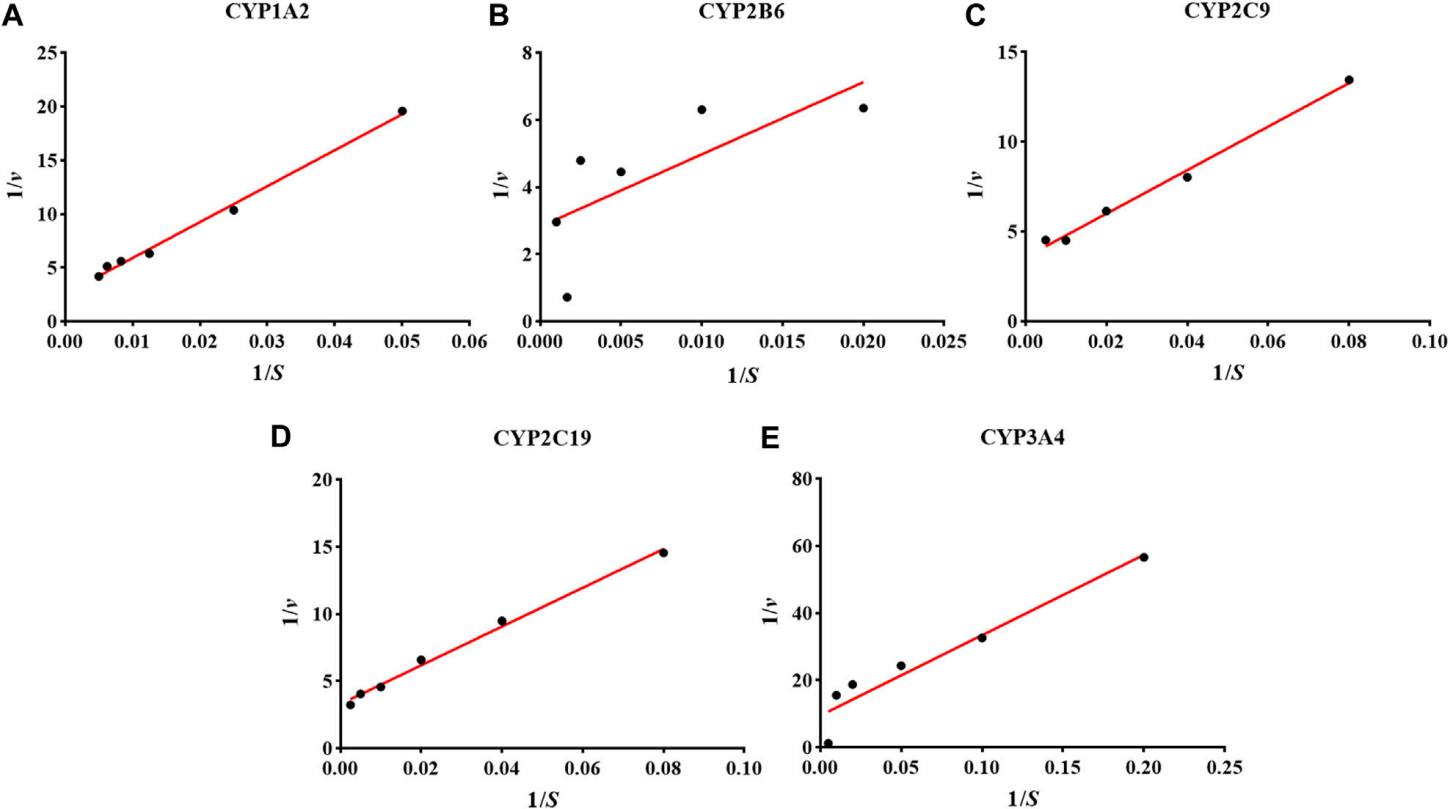
The results of the Lineweaver-Burk plot of CYP1A2 **(A)**, CYP2B6 **(B)**, CYP2C9 **(C)**, CYP2C19 **(D)**, and CYP3A4 **(E)** in the hiHeps model.

The K_m_ could reflect the catalytic ability of the metabolic enzyme, and also provide the necessary reference value of the probe drug for the evaluation system of CYP enzyme activity *in vitro* (Hakkola et al., 2020; Kong et al., 2020). We used the Lineweaver-Burk plot of CYP1A2 1), CYP2B6 2), CYP2C9 3), CYP2C19 4), and CYP3A4 5) in a hiHeps model to reveal the enzyme kinetics (Figure 1). The K_m_ values of the five probe drugs were calculated as follows: 115.9 μM for PHE, 150.8 μM for BUP, 41.9 μM for DIC, 33.6 μM for MEP, and 13.9 μM for MID. According to the references, the K_m_ values of PHE, BUP, DIC, MEP, and MID were as follows: 1.7–152.0 μM, 67.0–168.0 μM, 3.4–52.0 μM, 13.0–35.0 μM, and 1.0–14.0 μM, respectively. All K_m_ values of probe drugs in the hiHeps model were within the scope of literature, which indicates that the activity of CYP1A2, CYP2B6, CYP2C9, CYP2C19, and CYP3A4 in this hiHeps model met the requirements of an evaluation system for the effect of drugs on the activity of CYP enzymes *in vitro*. Secondly, according to the measured K_m_ in this experiment and the requirements of the cocktail method for the evaluation system, the concentration near the K_m_ and as low as possible was selected as the final concentration of the probe drugs (Chen et al., 2020). Therefore, the concentrations of the five probe drugs were selected in this system as follows: 50 μM for PHE, 50 μM for BUP, 20 μM for DIC, 20 μM for MEP, and 10 μM for MID. Moreover, when the incubation time was less than 60 min, the consumption curves of each probe drug had good linearity (*r* > 0.95). Therefore, 60 min was chosen as the final incubation time for the evaluation system.

### Study on the Interaction of Probe Substrates

**FIGURE 2:**
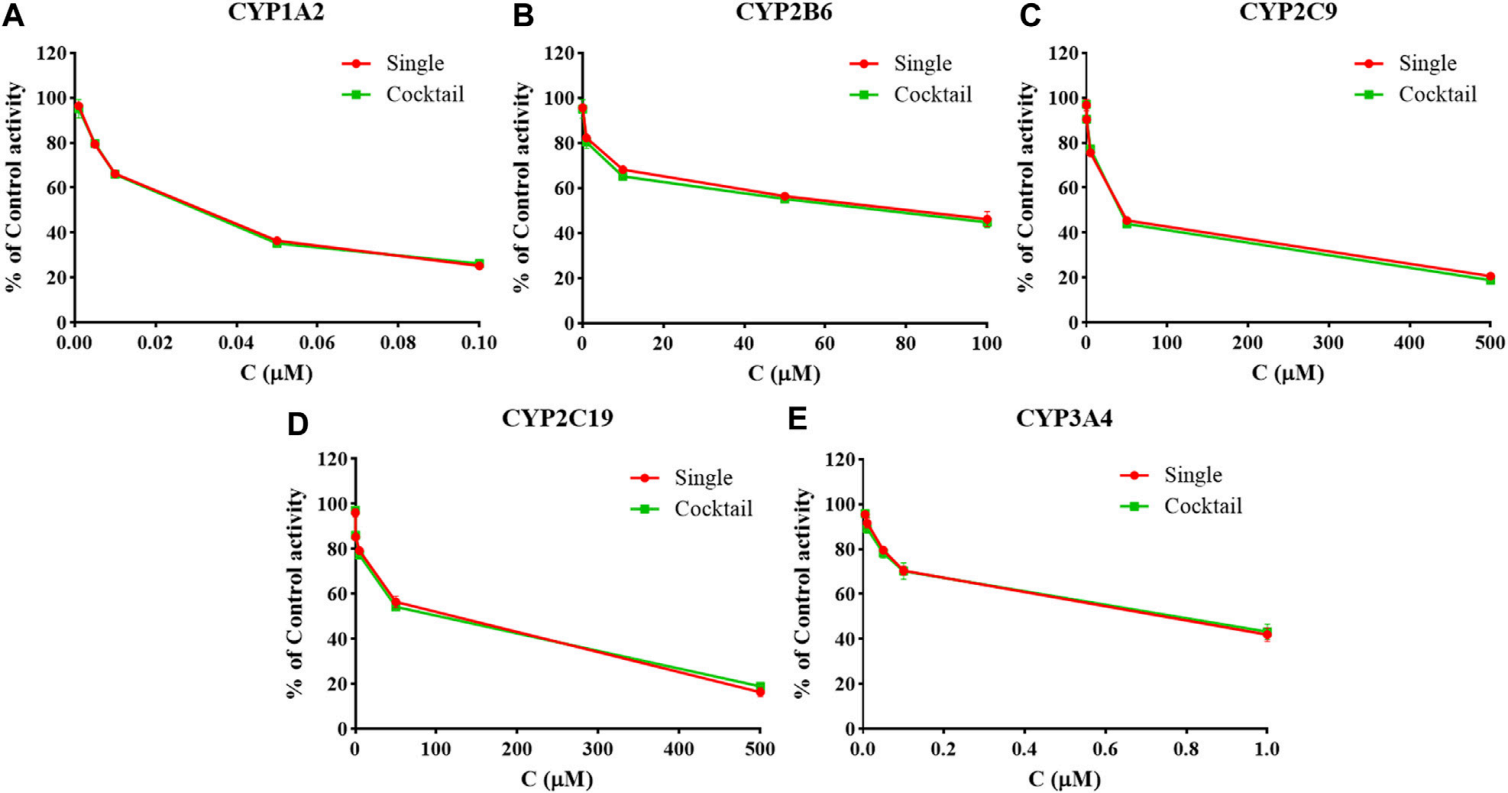
The activities of CYP1A2 **(A)**, CYP2B6 **(B)**, CYP2C9 **(C)**, CYP2C19 **(D)**, and CYP3A4 **(E)** in the hiHeps model between single probe substrates and cocktail probe substrates (mean ± SD, *n* = 3).

There was no significant difference in the activities of the five CYP enzymes in the hiHeps model between single probe substrates and cocktail probe substrates (*p* > 0.05) (Figure 2). The interaction between the probe substrates was not obvious under the conditions of substrate concentration and incubation time. Therefore, CYP enzyme activity in the evaluation system based on the hiHeps model could be rapid and detected by the cocktail method. A novel evaluation system for the effect of drugs on the activity of CYP enzymes *in vitro* based on hiHeps was successfully established.

### Comparison of Inhibitory Activity of Curcumin on Cytochrome P450 Enzymes in Three Models

**FIGURE 3:**
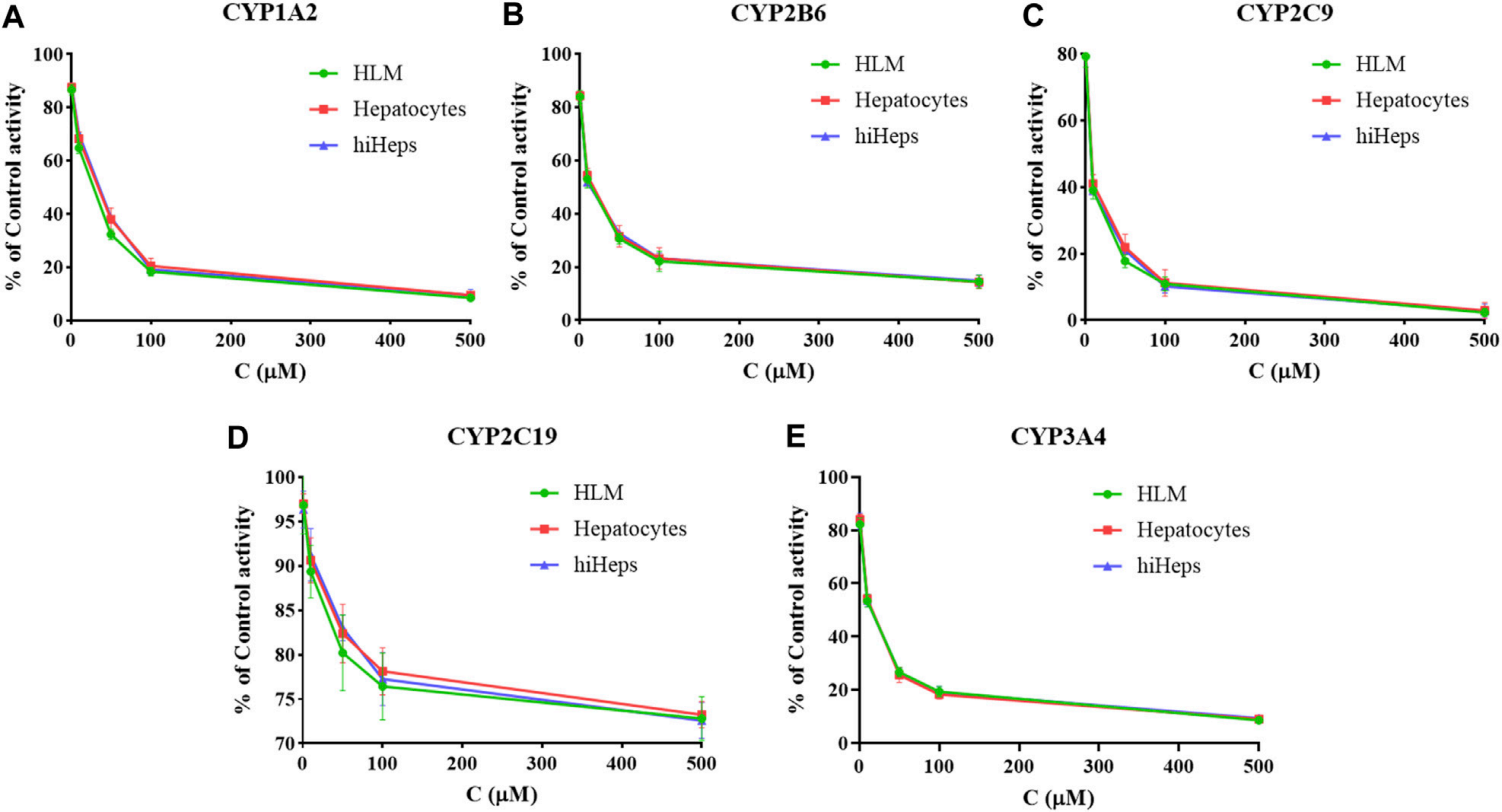
The inhibition curves of curcumin on CYP1A2 **(A)**, CYP2B6 **(B)**, CYP2C9 **(C)**, CYP2C19 **(D)**, and CYP3A4 **(E)** in HLM, hepatocytes, and hiHeps groups (mean ± SD, *n* = 3).

The inhibition curves of curcumin on the five CYP enzymes in HLM, hepatocytes, and hiHeps groups are shown in Figure 3, and the IC_50_ values of curcumin on the five CYP enzymes were compared among HLM, hepatocytes, and hiHeps groups (Table 1). In the hiHeps group, the IC_50_ value of curcumin on the CYP1A2 isoenzyme was 68.55 ± 4.89 μM, which indicated that curcumin had a weak inhibitory effect on the CYP1A2 isoenzyme. The IC_50_ value of curcumin on CYP2B6 and CYP3A4 was 35.96 ± 1.57 μM and 21.91 ± 1.76 μM, both were less than 50 μM, which belonged to the range of moderate inhibitory effect. The IC_50_ value of curcumin on CYP2C9 was 9.91 ± 1.12 μM suggesting that curcumin had a strong inhibitory effect on CYP2C9, and curcumin had no significant inhibitory effect on CYP2C19 (IC_50_ > 500 μM). In the HLM group, the IC_50_ values of curcumin on CYP1A2, CYP2B6, CYP2C9, CYP2C19, and CYP3A4 were 50.10 ± 2.71 μM, 30.00 ± 2.09 μM, 8.70 ± 1.36 μM, >500 μM, and 21.00 ± 1.27 μM, respectively. In the hepatocytes group, the IC_50_ values of curcumin on CYP1A2, CYP2B6, CYP2C9, CYP2C19, and CYP3A4 were 67.13 ± 3.81 μM, 37.72 ± 2.97 μM, 10.77 ± 1.43 μM, >500 μM, and 22.09 ± 1.63 μM, respectively.

**TABLE 1:** The IC_50_ values of curcumin to five CYP enzymes (*n* = 3).

CYP enzymes	IC_50_ (μM)		
	HLM	Hepatocytes	hiHeps
1A2	50.10 ± 2.71	67.13 ± 3.81	68.55 ± 4.89
2B6	30.00 ± 2.09	37.72 ± 2.97	35.96 ± 1.57
2C9	8.70 ± 1.36	10.77 ± 1.43	9.91 ± 1.12
2C19	>500	>500	>500
3A4	21.00 ± 1.27	22.09 ± 1.63	21.91 ± 1.76

The IC_50_ values in the HLM group were generally lower than that in the hepatocytes group and hiHeps group. This might be due to the direct exposure of enzyme proteins in HLM to inhibitors, while most enzyme proteins in the cell model were in the endoplasmic reticulum, and the inhibitors needed transmembrane transport to make contact with enzyme proteins. It suggested that there were differences in function of CYP enzymes between HLM and human liver tissue. The results showed that the activities of CYP1A2, CYP2B6, CYP2C9, CYP2C19, and CYP3A4 in the hiHeps group were similar to that in the hepatocytes group. Detection results of the hiHeps model were stable and reliable, which could be used to evaluate the effect of drugs on the activity of CYP enzymes *in vitro*. Due to rapid cell passage and good stability of CYP enzymes in hiHeps, the established evaluation system will become a powerful tool for the research of the effect of drugs on the activity of CYP enzymes *in vitro*, which has broad application prospects in drug research.

### Effect of NS, SF, and CNS on the Activity of Cytochrome P450 Enzymes *in vitro*

**FIGURE 4:**
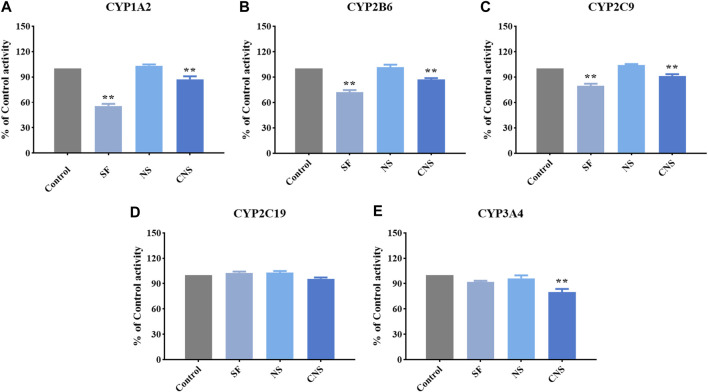
Effects of CNS, NS, and SF on the activity of CYP1A2 **(A)**, CYP2B6 **(B)**, CYP2C9 **(C)**, CYP2C19 **(D)**, and CYP3A4 **(E)** (mean ± SD, *n* = 3, **p < 0.01).

We assessed the effects of CNS, NS, and SF on the activity of CYP1A2, CYP2B6, CYP2C9, CYP2C19, and CYP3A4 (Figure 4 and Table 2). The IC_50_ values of NS on CYP1A2, CYP2B6, CYP2C9, CYP2C19, and CYP3A4 were 103.33 ± 1.53 μM, 101.67 ± 3.05 μM, 104.33 ± 1.15 μM, 102.83 ± 2.02 μM, and 96.21 ± 3.51 μM, respectively. The results suggested that NS had no significant effects on CYP1A2, CYP2B6, CYP2C9, CYP2C19, and CYP3A4 (*p* > 0.05). The IC_50_ values of SF on CYP1A2, CYP2B6, CYP2C9, CYP2C19, and CYP3A4 were 55.67 ± 2.52 μM, 72.00 ± 2.65 μM, 79.67 ± 2.52 μM, 102.67 ± 1.53 μM, and 91.82 ± 1.50 μM, respectively, which indicated that SF had significant inhibitory effects on CYP1A2, CYP2B6, and CYP2C9 enzymes (*p* < 0.05), but had no significant effects on CYP2C19 and CYP3A4 enzymes (*p* > 0.05). CNS had significant inhibitory effects on CYP1A2 (IC_50_ = 68.55 ± 4.89 μM), CYP2B6 (IC_50_ = 87.33 ± 1.53 μM), CYP2C9 (IC_50_ = 91.33 ± 2.08 μM), and CYP3A4 (IC_50_ = 80.11 ± 3.54 μM) (*p* < 0.05), but had no significant effect on the activity of CYP2C19 (IC_50_ = 95.67 ± 1.53 μM) (*p* > 0.05).

**TABLE 2:** Percentage of activity of five CYP450 subtypes in hiHeps groups.

CYP enzymes	Active percentage (%)		
	SF	NS	CNS
1A2	55.67 ± 2.52^b^	103.33 ± 1.53	68.55 ± 4.89^b^
2B6	72.00 ± 2.65^b^	101.67 ± 3.05	87.33 ± 1.53^b^
2C9	79.67 ± 2.52^b^	104.33 ± 1.15	91.33 ± 2.08^b^
2C19	102.67 ± 1.53	102.83 ± 2.02	95.67 ± 1.53
3A4	91.82 ± 1.50	96.21 ± 3.51	80.11 ± 3.54^b^

NS: notoginseng total saponins; SF: safflower total flavonoids; CNS: herb pair of notoginseng–safflower. Compared to control group.

a *p* < 0.05.

b *p* < 0.01.

CYP3A4 is the most predominant CYP enzyme and metabolizes approximately 50% of market drugs (Mann, 2006). Human CYP3A4 and CYP3A5 were the major enzymes in charge of the metabolism of 20(S)-protopanaxadiol or 20(S)-protopanaxatriol in NS (Hu et al., 2013). SF and NS alone had no significant effect on the activity of CYP3A4, but CNS (the compatibility form of SF and NS) significantly reduced the percentage of CYP3A4 enzyme activity, which showed an obvious inhibitory effect (*p* < 0.05). Therefore, the combined administration of SF and NS was beneficial to reduce the phase I metabolism of ginsenosides mediated by CYP3A4, and increase the exposure levels of the main bioactive components of NS *in vivo*. Compared with NS, the activity percentages of CYP1A2, CYP2B6, CYP2C9, and CYP3A4 were significantly decreased by CNS, which was of benefit for reducing the metabolism and enhancing the exposure levels of the main bioactive components in NS by CYP isozymes, and improved the exposure levels *in vivo*. The CYP2C subfamily make up about 20% of the total content of hepatic CYPs and metabolize about 20% of market drugs (Isvoran et al., 2017). SF, NS, and CNS had no significant effect on the activity of CYP2C19, however, CNS significantly reduced the activity percentage of CYP2C19 compared with SF and NS (*p* < 0.05). The results suggested that CNS reduced the phase I metabolism of saponins and flavonoids mediated by CYP2C19, and thus increased the exposure levels of the main bioactive ingredients of SF and NS *in vivo*.

This result was consistent with the experimental data *in vivo*, which verified the reliability and stability of the evaluation system. Moreover, the mechanism of effective compatibility of NS and SF based on a phase I metabolic enzyme was elucidated from *in vitro* experiments on the basis of the hiHeps model. In addition, the ability of hiHeps in evaluating the induced effect of drugs on CYP enzymes was found in this research. The application of hiHeps in metabolism research will continue to be improved in the follow-up study.

## Conclusion

This research established a new evaluation system for the effect of drugs on CYP enzymes *in vitro* based on hiHeps. The validity and stability of the evaluation system were verified by comparing those of HLM and human hepatocytes. Subsequently, the system was applied to evaluate the inhibition effect of NS, SF, and CNS on the activity of CYP enzymes, which explained the mechanism of effective compatibility of NS and SF based on CYP enzymes. This study provided new methods and ideas for drug safety/toxicity research in preclinical and clinical stages.

## Data Availability

The original contributions presented in the study are included in the article/Supplementary Material, further inquiries can be directed to the corresponding authors.
