# Supplementary material for: A Novel System for Evaluating the Inhibition Effect of Drugs on Cytochrome P450 Enzymes in vitro Based on Human-Induced Hepatocytes (hiHeps)
Source: Front Pharmacol. 2021 Oct 28;12:748658. doi: 10.3389/fphar.2021.748658 (PMC8580884; doi:10.3389/fphar.2021.748658)
Supplement: Supplementary file 1 [file DataSheet1.DOCX]

*Supplementary Material*


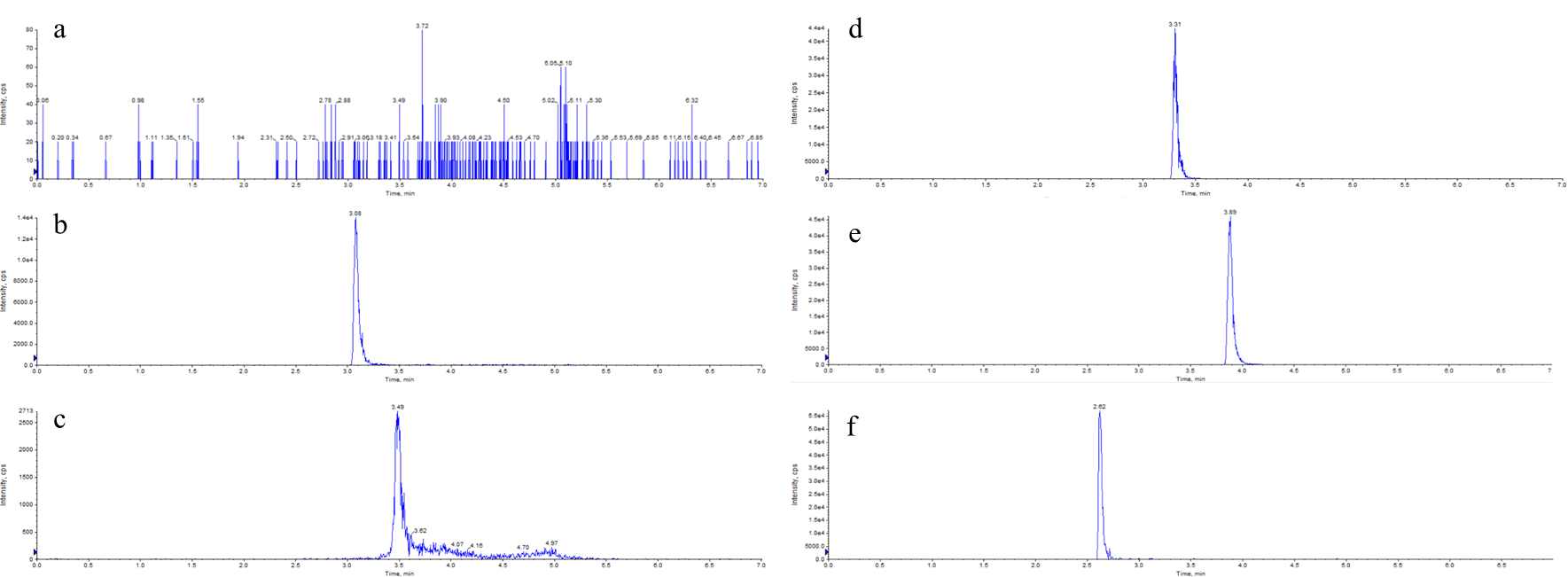


Figure S1. Representative UPLC-MS/MS chromatograms of (a) blank sample; (b) acetaminophen; (c) Hydroxybupropion; (d) 4'-Hydroxymephenytoin; (e) 4'-Hydroxydiclofenac; and (f) 1'-Hydroxymidazolam

Table S1. The MS parameters of probe drugs and corresponding metabolites

| Analyte | Q1 (*m*/*z*) | Q2 (*m*/*z*) | DP (V) | CE (V) |
| --- | --- | --- | --- | --- |
| Phenacetin | 180.2 | 110.0 | 72 | 28 |
| Acetaminophen | 152.2 | 111.2 | 80 | 25 |
| Bupropion | 241.1 | 185.1 | 71 | 18 |
| Hydroxybupropion | 238.2 | 138.9 | 107 | 32 |
| Diclofenac | 295.6 | 249.9 | 61 | 20 |
| 4'-Hydroxydiclofenac | 313.6 | 204.7 | 82 | 27 |
| (*S*)-Mephenytoin | 219.3 | 134.3 | 91 | 24 |
| 4'-Hydroxymephenytoin | 235.3 | 150.2 | 108 | 24 |
| Midazolam | 326.1 | 291.1 | 91 | 36 |
| 1'-Hydroxymidazolam | 342.1 | 323.9 | 112 | 31 |
| Carbamazepine | 237.0 | 193.9 | 32 | 28 |

Table S2. The standard curve, linear range, correlation coefficient and [lower limit of quantitation](https://www.ncbi.nlm.nih.gov/pubmed/29391006) (LLOQ) for each metabolite of probe substrate

| Analyte | Standard curves | Linear range  (ng/mL) | Correlation coefficient (r) | LLOQ  (ng/mL) |
| --- | --- | --- | --- | --- |
| Acetaminophen | y=0.0014x+0.00076 | 1-200 | 0.9976 | 1 |
| Hydroxybupropion | y=0.00013x-0.00046 | 1-200 | 0.9945 | 1 |
| 4'-Hydroxydiclofenac | y=0.00026x+0.00076 | 1-200 | 0.9936 | 1 |
| 4'-Hydroxymephenytoin | y=0.00076x-0.00042 | 1-200 | 0.9957 | 1 |
| 1'-Hydroxymidazolam | y=0.00014x+0.00013 | 1-200 | 0.9989 | 1 |

Table S3. Intra-/inter-day precision and accuracy of five probe drugs (mean ± SD, n = 6)

| Analyte | QC conc.  (ng/mL) | Intra-day | | | Inter-day | | |
| --- | --- | --- | --- | --- | --- | --- | --- |
|  |  | Calc. Conc.  (ng/mL) | Precision  RSD (%) | Accurancy  (%) | Calc. Conc.  (ng/mL) | Precision  RSD (%) | Accurancy  (%) |
| Acetaminophen | 1 | 1.02±0.06 | 3.43 | 98.55±3.45 | 1.01±0.02 | 2.42 | 102.13±3.77 |
|  | 50 | 50.46±1.21 | 2.29 | 100.53±2.18 | 51.07±2.64 | 3.11 | 100.79±2,76 |
|  | 200 | 203.57±2.53 | 2.63 | 100.57±1.26 | 204.34±4.26 | 2.96 | 100.26±2.16 |
| Hydroxybupropion | 1 | 1.04±0.07 | 3.62 | 102.54±1.22 | 0.96±0.11 | 4.57 | 97.21±5.64 |
|  | 50 | 50.22±2.31 | 3.68 | 100.12±2.66 | 50.70±1.67 | 1.68 | 100.54±2.88 |
|  | 200 | 203.32±2.96 | 1.65 | 101.44±2.63 | 198.43±2.15 | 2.26 | 99.23±1.78 |
| 4'-Hydroxydiclofenac | 1 | 1.01±0.02 | 1.01 | 100.32±1.26 | 1.02±0.03 | 2.06 | 100.63±2.16 |
|  | 50 | 51.62±1.24 | 1.25 | 100.22±1.77 | 50.26±1.27 | 2.52 | 100.29±1.75 |
|  | 200 | 203.15±2.57 | 2.48 | 100.96±3.14 | 205.27±3.16 | 2.62 | 101.02±2.98 |
| 4'-Hydroxymephenytoin | 1 | 1.04±0.04 | 2.77 | 100.89±2.01 | 1.05±0.04 | 3.25 | 101.23±2.42 |
|  | 50 | 52.88±3.21 | 2.76 | 101.24±2.79 | 54.21±2.26 | 4.67 | 102.06±3.73 |
|  | 200 | 204.37±6.97 | 5.21 | 101.72±4.46 | 201.14±4.89 | 3.65 | 100.14±3.26 |
| 1'-Hydroxymidazolam | 1 | 1.04±0.03 | 2.14 | 100.17±2.25 | 1.01±0.02 | 1.24 | 100.31±1.76 |
|  | 50 | 52.14±1.32 | 1.87 | 100.68±1.62 | 51.67±1.86 | 1.99 | 100.82±2.03 |
|  | 200 | 205.20±4.13 | 2.14 | 100.95±2.54 | 203.16±3.33 | 2.18 | 101.08±2.36 |

Table S4. Recovery and matrix effect of five probe drugs (mean ± SD, n = 6)

| Compound | QC conc. (ng/mL) | Matrix effect (%) | Recovery (%) |
| --- | --- | --- | --- |
| Acetaminophen | 1 | 94.53±1.80 | 92.15±0.27 |
|  | 50 | 89.55±1.40 | 91.44±1.04 |
|  | 200 | 86.47±2.65 | 95.78±2.04 |
| Hydroxybupropion | 1 | 82.45±3.82 | 86.36±0.91 |
|  | 50 | 80.33±1.97 | 89.25±1.18 |
|  | 200 | 78.59±1.91 | 88.44±2.20 |
| 4'-Hydroxydiclofenac | 1 | 95.11±2.67 | 96.14±0.55 |
|  | 50 | 94.24±1.63 | 96.47±0.94 |
|  | 200 | 94.48±1.12 | 91.88±2.08 |
| 4'-Hydroxymephenytoin | 1 | 91.25±0.97 | 93.26±0.63 |
|  | 50 | 88.26±1.10 | 97.27±0.82 |
|  | 200 | 84.11±0.82 | 95.94±2.74 |
| 1'-Hydroxymidazolam | 1 | 98.26±0.13 | 94.18±0.39 |
|  | 50 | 96.47±0.30 | 95.74±1.27 |
|  | 200 | 95.38±0.81 | 91.28±1.62 |
